# Novel insights into obesity and diabetes through genome-scale metabolic modeling

**DOI:** 10.3389/fphys.2013.00092

**Published:** 2013-04-25

**Authors:** Leif Väremo, Intawat Nookaew, Jens Nielsen

**Affiliations:** Systems and Synthetic Biology, Department of Chemical and Biological Engineering, Chalmers University of TechnologyGothenburg, Sweden

**Keywords:** systems biology, metabolism, obesity, diabetes, genome-scale metabolic models, metabolic networks, topology, constraint-based modeling

## Abstract

The growing prevalence of metabolic diseases, such as obesity and diabetes, are putting a high strain on global healthcare systems as well as increasing the demand for efficient treatment strategies. More than 360 million people worldwide are suffering from type 2 diabetes (T2D) and, with the current trends, the projection is that 10% of the global adult population will be affected by 2030. In light of the systemic properties of metabolic diseases as well as the interconnected nature of metabolism, it is necessary to begin taking a holistic approach to study these diseases. Human genome-scale metabolic models (GEMs) are topological and mathematical representations of cell metabolism and have proven to be valuable tools in the area of systems biology. Successful applications of GEMs include the process of gaining further biological and mechanistic understanding of diseases, finding potential biomarkers, and identifying new drug targets. This review will focus on the modeling of human metabolism in the field of obesity and diabetes, showing its vast range of applications of clinical importance as well as point out future challenges.

## Introduction

Metabolism, the set of chemical reactions taking place in a cell, plays an important part in maintaining cell functionality by responding to various perturbations, sustaining the production of essential molecules and cell components, breaking down compounds for energy production and catabolizing xenobiotics. The drivers behind these typically thousands of reactions are enzymes, whose concentrations are controlled by several levels of regulation, affected both by the cell type and its surrounding environment. Due to the central role of metabolism it is not surprising that its dysfunction is associated with several complex diseases, of which prominent examples are obesity and diabetes. The severity of this is further emphasized by the fact that more than 360 million people are suffering from type 2 diabetes (T2D) and that in 2011 this disease contributed to more than 3.5 million deaths in middle-income countries (Whiting et al., 2011; Scully, 2012). The prevalence of T2D has not stopped increasing and the incidence among young adults and children is rising for each decade (Chen et al., 2012). In 2030, it is projected that 552 million people, corresponding to 10% of the global adult population, will suffer from T2D (Whiting et al., 2011).

T2D is characterized by impaired insulin secretion and insulin resistance, mainly manifested in liver, skeletal muscle, and adipose tissue, leading to malfunctioning glucose homeostasis and metabolism. This complex metabolic disease is associated with a multitude of risk factors including physical inactivity, diet, gut microbiome composition, obesity, aging and genetic components (Doria et al., 2008). The cellular events characteristic of T2D and obesity, but also influenced by different treatment strategies (including physical activity, diet, calorie restriction, and surgery), are eventually manifested through changes in metabolism. It is of course highly relevant to elucidate mechanistic explanations for the disease, as well as for existing treatments, in order to gain further biological understanding, identify new biomarkers, and find potential drug targets. Due to the interconnected nature of metabolism and the fact that a cell acts as a system, it is logical to take a holistic approach to investigate these issues.

In this context, genome-scale metabolic models (GEMs) that are *in silico* representations of metabolism at the genome level, have emerged as a key tool in the field of systems biology (Mardinoglu et al., 2013b). Further on, the increasing generation and availability of high-throughput omics data (e.g., transcriptomics or proteomics) is not only pushing the need of GEMs as well as enabling advanced analysis (Patil and Nielsen, 2005; Yizhak et al., 2010), but is also driving improvements in the reconstruction process itself (Shlomi et al., 2008; Agren et al., 2012). Several authors have reviewed human GEMs and their growing scope of applications in general (Bordbar and Palsson, 2012; Mardinoglu and Nielsen, 2012). In this review we focus specifically on the modeling of human metabolism in the field of obesity and diabetes.

## Human genome-scale metabolic models

Historically, GEMs were initially developed to study microbial metabolism, starting with the reconstruction of *Haemophilus influenza* metabolism (Edwards and Palsson, 1999). Since then, GEMs for many pathogens and industrially relevant organisms have been produced (Oberhardt et al., 2009). With a shift in focus to human metabolism, early attempts to human GEMs include the mitochondrial metabolic network (Vo et al., 2004). In 2007, two global human metabolic network reconstructions, Recon 1 (Duarte et al., 2007) and the Edinburgh Human Metabolic Network (EHMN) (Ma et al., 2007), were published. The EHMN was later updated with information about cellular compartments (Hao et al., 2010). In 2012, the Human Metabolic Reaction (HMR) database was created (Agren et al., 2012), encompassing information from Recon 1 and EHMN, as well as from the Kyoto Encyclopedia of Genes and Genomes (KEGG) database (Kanehisa et al., 2012), and later updated with extensive lipid metabolism (Mardinoglu et al., 2013a). In 2013, the metabolic reconstruction Recon 2 was published (Thiele et al., 2013). These generic human GEMs have been shown to have many applications, including e.g., the study of disease comorbidity (Lee et al., 2008), cancer drug target discovery (Agren et al., 2012; Jerby and Ruppin, 2012), biomarkers for inborn errors of metabolism (Shlomi et al., 2009) and brain energy metabolism in Alzheimer's disease (Lewis et al., 2010).

### The structure of GEMs

**Figure 1:**
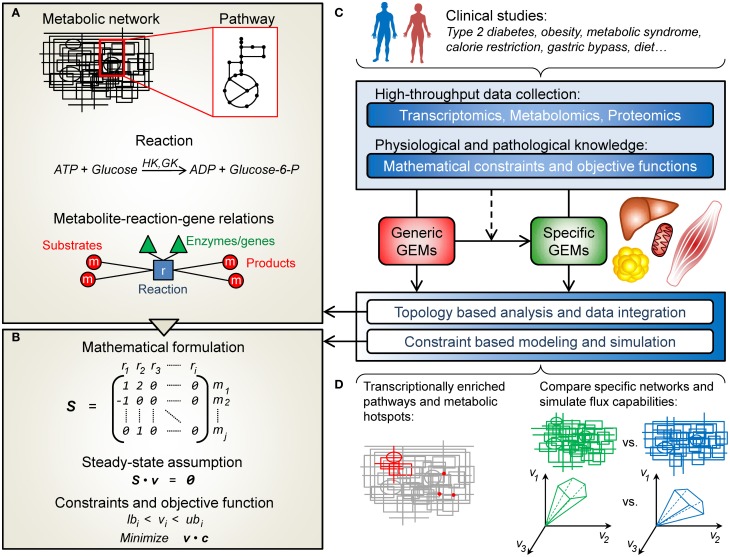
**An overview of human genome-scale metabolic models (GEMs) and their applications in the field of obesity and diabetes. (A)** A metabolic network is in simple terms a list of the chemical reactions taking place in a cell. These reactions can be grouped into pathways and associated with a particular cellular compartment (e.g., mitochondria). Metabolites can be passed between compartments through transport reactions. Each reaction can be associated to its corresponding enzyme-coding genes, and together all the reactions provide a network structure connecting metabolites, reactions and genes. **(B)** The metabolic network can be represented mathematically by the stoichiometric matrix, *S*, containing the stoichiometric coefficients of the metabolites (rows) taking part in each reaction (columns). Under the constraint based modeling framework it is assumed that the metabolite concentrations are unchanged (*Sv* = 0). Further on, additional constraints can be put on the flux vector, *v*, to find capable and probable flux distributions. Alternatively, flux balance analysis (FBA) can be used to find a flux vector that optimizes an objective function (e.g., maximize ATP production). **(C)** GEMs have been used to study obesity- and diabetes-related conditions. Clinical data can be used to construct context specific GEMs from generic ones. This type of data can also be integrated and analyzed, in combination or separately, with the GEMs, in a topological or simulation based manner. **(D)** This enables e.g., the identification of transcriptionally affected reactions and pathways as well as metabolic hotspots, or the comparison of simulation results in terms of network capabilities.

The conceptual structure of a GEM is summarized in Figure 1A. In its simplest form a GEM is a list of mass-balanced reactions, describing the conversion of substrate metabolites into product metabolites. In addition, reactions can be associated to cellular compartments (e.g., cytoplasm or mitochondria), thus partitioning the metabolic network into sections connected only through transport reactions. When the information is available, enzyme-coding genes are associated with their corresponding reactions. As such, the GEM constitutes a knowledge-base of human metabolism and its information along with the provided network topology can be used to analyze and interpret external high-throughput data. Apart from this, GEMs can be used for simulating how metabolism operates at different conditions using the constraint-based modeling framework described next.

### Constraint-based modeling and simulation

Constraint-based modeling is used to simulate and predict metabolic phenotypes (Bordbar and Palsson, 2012). In order to do this, the GEM is expressed in mathematical terms in form of a stoichiometric matrix, *S*, where rows correspond to metabolites and columns represent reactions (Figure 1B). For a particular reaction, or column of *S*, the metabolites taking part in the reaction are denoted with their stoichiometric coefficient (negative for substrates and positive for products), all other values are set to zero. Under the constraint-based modeling framework, it is assumed that the concentrations of all metabolites are constant, meaning that the sum of fluxes of the reactions where the metabolite is consumed has to be equal to the sum of fluxes of the reactions where the metabolite is produced. Mathematically, this can be written as *Sv* = 0, where *v* is a vector of fluxes for each reaction. This system is underdetermined, i.e., there are many possible flux vectors that solve the system. However, the solution space can be shrunk by applying constraints on *v*, based on current physiochemical and biological knowledge. A common approach is to use linear programming to find a flux vector that optimizes an objective function (e.g., to maximize a specific flux), given the constraints on *v* and the condition *Sv* = 0. This approach is called flux balance analysis, FBA (Orth et al., 2010).

### Cell-type specific GEMs

Generic GEMs (such as Recon 1, EHMN and HMR) are also useful resources for new reconstructions, in particular when considering cell-type specific networks. Manual reconstruction is very laborious and because of this much interest has been put into algorithmic approaches for automated or semi-automated GEM reconstruction (Shlomi et al., 2008; Blazier and Papin, 2012). An example of this is the INIT algorithm (Agren et al., 2012), and the GIMME algorithm (Becker and Palsson, 2008), which has been used in studies included in this review and is described briefly in the next section. In general these kinds of algorithms start with a generic GEM, representing the superset of human metabolism, and based on cell or tissue specific omics data (e.g., transcriptomics or proteomics) the metabolic network is reduced into a subset of reactions, representing the metabolic capabilities of a specific cell type. These cell-type specific networks can then be used for analysis of metabolic features of specific cell types as well as analyzing differences in metabolism, e.g., between a healthy cell and a cancer cell.

## Studying obesity and diabetes using human GEMs

Falling within the scope of this review, 10 studies using GEMs in the area of obesity and diabetes were identified and are summarized in Table 1. High-throughput omics data (gene expression, proteomics, and metabolite profiles) as well as physiological and pathological knowledge can be integrated, in combination or separately, with GEMs in order to unravel the underlying mechanisms of obesity and diabetes (Figure 1C). This information has also been used to construct context-specific metabolic networks, better representing the key cell types involved in these diseases: adipocytes (fat cells), hepatocytes (liver cells), and myocytes (skeletal muscle cells). Roughly, the following studies have either used network topology to contextualize omics data or used specific GEMs to simulate and evaluate flux capabilities (Figure 1D).

**Table 1:** **Overview of studies using human GEMs to study obesity and diabetes**.

**References**	**Topic/condition**	**Tissue(s)**	**Source network(s)**	**Modified network(s)**
**TOPOLOGY BASED ANALYSIS AND DATA INTEGRATION**				
Duarte et al., 2007	Gastric bypass surgery	Skeletal muscle	Recon 1	No modification
Capel et al., 2009	Calorie restriction	Adipose tissue	Recon 1, EHMN	No modification
Deo et al., 2010	Impaired glucose tolerance	Blood	Recon 1	Metabolic reaction network (MRN)
Zelezniak et al., 2010	Type 2 diabetes	Skeletal muscle	Recon 1, EHMN	No modification
Mutch et al., 2011	Calorie restriction	Adipose tissue	EHMN	No modification
Morine et al., 2011	Metabolic syndrome and n3 PUFA diet	Adipose tissue	EHMN	Transformation to an enzyme-centric network
**CONSTRAINT BASED MODELING AND SIMULATION**				
Thiele et al., 2005	Diabetes	Heart	Mitochondrial	Extended mitochondrial
Becker and Palsson, 2008	Gastric bypass, glucose/insulin infusion, obesity	Skeletal muscle	Recon 1	Myocytes in different conditions
Bordbar et al., 2011	Type 2 diabetes	Liver, skeletal muscle, adipose tissue	Recon 1	Hepatocyte, myocyte, adipocyte
Mardinoglu et al., 2013a	Obesity	Adipose tissue	HMR	Adipocyte

### Exploiting metabolic network topology—putting content into context

Topological analysis of the generic human metabolic networks Recon 1 and EHMN has been exploited to dissect the complexity of metabolism in many studies. In fact, one of the first applications of Recon 1 was to map human skeletal muscle gene expression data on to the network, in order to interpret the metabolic effects of gastric bypass surgery (Duarte et al., 2007). Both surgically induced calorie restriction, as in this example, and calorie-restricted diet, as in several examples below, are used as weight loss treatment for obesity. Weight loss in obese individuals, in turn, is known to improve insulin sensitivity and to have a positive effect on metabolism (Reaven, 2005). In their study Duarte et al. (2007) found that anaerobic metabolism was up-regulated and that oxidative phosphorylation was down-regulated one year after surgery, suggesting that the effects of calorie restriction remain even after weight stabilization.

An alternative way to interpret transcriptome data is to shift the focus from reactions to metabolites. Patil and Nielsen (2005) described one such algorithm which uses the gene-enzyme-reaction-metabolite relationship and scores each metabolite by combining the *p*-values of its connected genes, based on gene expression data. This enables the identification of what the authors refer to as reporter metabolites, i.e., metabolic hotspots or metabolites around which important transcriptional changes occur.

Capel et al. (2009) used reporter metabolite analysis to detect differences in adipose tissue of obese subjects undergoing a dietary intervention program (calorie restriction followed by weight stabilization). The authors used both Recon 1 and EHMN to define the gene-metabolite connections and identified acyl-CoA, CoA, acetyl-CoA, NADP^+^/NADPH, and ubiquinol/ubiquinone as reporter metabolites, surrounded by down-regulated enzymes during calorie restriction and showing a reversed pattern during weight stabilization. In a similar study the EHMN was used to identify reporter metabolites as candidate biomarkers for successful weight maintenance. This was done by comparing adipose tissue gene expression of subjects maintaining weight with those regaining weight, after undergoing calorie restriction (Mutch et al., 2011). Reporter metabolites for the weight maintainers, during calorie restriction, were in agreement with the previous study by Capel et al. (2009). In particular the weight maintainers showed a more coordinate response to calorie restriction, compared to weight regainers, shown by a highly connected sub-network of significant enzymes and metabolites.

Zelezniak et al. (2010) used reporter metabolite analysis together with Recon 1 and EHMN to find metabolic signatures of T2D in skeletal muscle, using two publically available gene expression datasets. Metabolites from the TCA cycle, oxidative phosphorylation and lipid metabolism were identified as reporter metabolites, several of these being potential novel biomarkers for T2D. Further on, for the genes associated to the reporter metabolites, the authors found enrichment of transcription factor binding motifs belonging to the CREB, NRF1, and PPAR families.

In order to investigate the effect of n-3 polyunsaturated fatty acid (PUFA) intake on adipose tissue gene expression among subjects with metabolic syndrome, Morine et al. (2011) converted the EHMN to an enzyme-centric directed network, so that two enzymes were connected if a product metabolite of the first enzyme was used as a substrate by the second. Further on, by determining the co-expression of each pair of connected enzymes, the authors were able to identify metabolically feasible co-expressed paths (i.e., paths of connected metabolite conversions, where each conversion is co-expressed with the next one in the sequence). In particular, co-expressed paths containing enzymes whose expression was affected by n-3 PUFA intake were identified and inversely correlated to a urinary biomarker of oxidative stress. Their approach also identified pathway overlap in the metabolism of inositol phosphate derivatives, lipids and fatty acids, important in the context of dietary induced signaling cascades.

The previously mentioned studies used GEMs to analyze transcriptome data, however, other high-throughput data can also be contextualized with the aid of metabolic networks. Deo et al. (2010) analyzed metabolite profiles from blood of subjects with normal (NGT) and impaired glucose tolerance (IGT) undergoing an oral glucose tolerance test (OGTT). The authors constructed a so called metabolic reaction network, MRN, based on Recon 1 and 737 additional transport reactions. In MRN, metabolites are nodes and two metabolites are connected if they take part in the same enzymatic or transport reaction. Integrating the MRN with the metabolite data, the authors were able to identify active subnetworks, i.e., metabolic regions affected by changing metabolite levels during an OGTT. Using tissue specific GEMs (Shlomi et al., 2008) they found that the subnetworks were active in kidney and liver metabolism. They also point out the importance of differences in solute carrier activities in response to glucose.

### Cell-type specific models and flux simulation

The examples in the previous section reflect how the topology of GEMs can be used to interpret high-throughput data in a metabolic context. Besides this, GEMs have also been applied as models for simulation of metabolic fluxes in the study of diabetes and obesity. An important application in this context is to use clinical data of exchange fluxes to predict intracellular metabolic fluxes.

One of the first studies using GEMs in the context of diabetes was that of Thiele et al. (2005). The authors used a previously published mitochondrial metabolic network of the human cardiomyocyte (Vo et al., 2004) and expanded it by including ketone body degradation, with the purpose to study the effects of diabetes and different diets. In order to simulate diabetic conditions, the authors applied metabolic constraints on the fluxes through selected exchange reactions based on experimental measurements (increased mitochondrial fatty acid uptake, reduced glucose uptake, and increased ketone body uptake). Given these constraints, the authors calculated possible solutions to *Sv* = 0, resulting in a set of flux vectors, or candidate metabolic network states, which enabled the authors to investigate capable and most probable fluxes through selected reactions. Their analysis showed that network stoichiometry, as opposed to active enzyme levels, could explain the reduced flux through mitochondrial pyruvate dehydrogenase in diabetes.

When modeling human metabolism it is of course important to consider cell-type specific metabolism. Becker and Palsson (2008) developed an algorithm, Gene Inactivity Moderated by Metabolism and Expression (GIMME), that uses gene expression data and a generic GEM, together with objective functions, to produce context-specific functional GEMs. Simply put, GIMME assumes that transcript levels correlate to reaction fluxes and thus removes reactions from the generic network where the corresponding enzymes show low expression. However, the new GEM is required to be able to perform certain characteristic metabolic functions (specified by the objective functions and FBA) and to ensure this, previously removed reactions are allowed to be reintroduced in a way that minimizes the deviation between the expression data and the resulting model. The authors used Recon 1 and GIMME to produce 42 context-specific models of human myocytes using three gene expression datasets (gastric bypass, glucose/insulin infusion, and obesity) and the metabolic requirement of at least sub-optimal ATP producing capabilities. They found that the metabolic networks of a patient before and after surgery or glucose/insulin infusion are more similar to each other than to networks of other patients. They also observed that patients after glucose/insulin infusion show higher ATP production, as expected.

Bordbar et al. (2011) used Recon 1 as a starting point to construct cell-type specific GEMs for adipocytes, hepatocytes, and myocytes and additionally connected these models by introducing a joint blood compartment. This multi-tissue GEM was used as input to GIMME, together with transcription data from adipose, liver, and skeletal muscle tissue of healthy obese and T2D obese subjects. The resulting context-specific models were used to compare the metabolic reaction activity of the two subject groups. Using this approach, the authors found several known differences between healthy obese and T2D obese subjects. They also identified cysteine dioxygenase, which was found inactive in the adipose of diabetic subjects, as a potential reason for elevated levels of cytotoxic cysteine and decreased levels of taurine, an important metabolite in diabetes and shown to reduce diabetic symptoms (Hansen, 2001).

Mardinoglu et al. (2013a) extended the HMR with extensive lipid metabolism and used it, together with adipocyte specific proteomics data (Uhlen et al., 2010), to construct a functional adipocyte GEM. Constraints on the fluxes were added from clinical data (fatty acid transport as well as glucose and amino acid uptake rates) from lean and obese subjects in order to simulate lipid droplet formation using FBA. The authors found that lean subjects show larger dynamics in lipid droplet formation compared to obese subjects. Further on, feasible and most probable metabolic flux distributions (given *Sv* = 0 and the constraints on *v*) were calculated using a random sampling algorithm (Bordel et al., 2010) in order to find reactions with changing fluxes between obese and lean subjects. By correlating these results with gene expression data from the same subjects, the authors were able to identify reactions likely to be transcriptionally regulated, including down-regulation of glucose and fatty acid uptake, oxidative phosphorylation, beta-oxidation, fatty acid metabolism, tricarboxylic acid cycle, and beta-alanine metabolism in obese subjects.

## Concluding remarks and future perspectives

Human GEMs have proven to be very valuable in studies of metabolic disorders, such as obesity and diabetes. Through compilation of years of community research the human GEMs encompass state of the art genome-wide metabolic knowledge. As such, they provide a biologically meaningful context for the interpretation of various high-throughput datasets. As shown in many of the studies discussed in this review, topology-based analysis can successfully identify results that are not evident from traditional analyses. An example of this is the study by Deo et al. (2010) where active metabolic regions could be identified using Recon 1, but not when applying traditional pathway enrichment methods.

One of the strengths of GEMs is their ability to generate new hypotheses and predictions, as shown in many of the previously mentioned studies. As for any modeling approach, validation of the results is of course essential. In particular, since GEMs are represented on a genome-wide scale they integrate well with omics data. This means that predictions made by the GEMs, such as reporter metabolites as candidate biomarkers, can be validated on transcript, protein as well as metabolite levels. In general, the previously mentioned studies tend to support their modeling results with evidence in the literature. A high correlation with experimental data is of course confirming GEMs as a relevant modeling approach. However, to strengthen the actual novel predictions the ambition should be to validate and confirm these through subsequent experiments, e.g., as in Frezza et al. (2011). With the wider use of GEMs there are likely to come many studies in the future that will evaluate model predictions.

Several challenges still remain in the area of human metabolic modeling. One is to improve constraint-based modeling techniques for human metabolism, in particular the issue of formulating valid metabolic objective functions that reflect healthy and disease phenotypes in a manner that leads to correct biological conclusions. Further on, the predictive power will always be limited by the content of the used GEM. Thus, advances in the reconstruction procedure, both of generic and specific GEMs, will be important in order to sustain the production of up-to-date networks. Since the pathology of obesity and diabetes is highly dependent of the interplay between liver, adipose and muscle tissue, the next step in metabolic modeling should be to focus on model integration, both in terms of capturing interactions of cell types within each tissue, as well as between tissues. First steps include the multi-tissue model (Bordbar et al., 2011) summarized in this review, as well as the metabolic interaction of different brain cells (Lewis et al., 2010), both which are encouraging for future developments. Finally, although metabolism is a major component of obesity and diabetes, it is important to consider the impact of additional biological processes. A long-term future direction should be to integrate different models (such as GEMs, signaling pathways, and gene regulatory networks) to better recapitulate these pathologies at multiple length and time scales. Promising conceptual work on integrating models includes the whole-cell model by Karr et al. (2012) and the pharmacokinetic model by Krauss et al. (2012).

### Conflict of interest statement

The authors declare that the research was conducted in the absence of any commercial or financial relationships that could be construed as a potential conflict of interest.
